# Dilemma on Pancreatic Uncinate Process Uptake on Ga68-DOTA Peptide PET/CT in Pediatric Neuroblastoma: Physiologic or Metastases?

**DOI:** 10.2174/0118744710226018250206105536

**Published:** 2025-02-17

**Authors:** Nedim C.M. Gülaldi, Nadide Basak Gülleroglu, Selma Cakmakci, Fatma Arzu Görtan, Neriman Sari

**Affiliations:** 1 Department of Nuclear Medicine, University of Health Sciences, Ankara Bilkent City Hospital, Ankara, Türkiye;; 2 Department of Pediatric Radiology, University of Health Sciences, Ankara Bilkent City Hospital, Ankara, Türkiye;; 3 Department of Pediatric Hematology-Oncology, The Ministry of Health, Ankara Bilkent City Hospital, Ankara, Türkiye;; 4 Department of Nuclear Medicine, The Ministry of Health, Ankara Bilkent City Hospital, Ankara, Türkiye;; 5 Department of Pediatric Hematology-Oncology, University of Health Sciences, Ankara Bilkent City Hospital, Ankara, Türkiye

**Keywords:** Neuroblastoma, Ga68-DOTA, PET/CT, pancreas, MIBG, MRI

## Abstract

**Introduction:**

The Ga68-DOTATATE PET/CT scan is an alternative imaging modality for the follow-up of children with neuroblastoma when the I123-MIBG scan was negative or weak. Somatostatin receptors (SSR) can be expressed in neuroblastoma lesions, and when this happens, targeting these receptors may be a good alternative to treating this disease in addition to conventional treatments. Our aim is to focus on the interpretation of one of the physiological tracer uptake sites, the uncinate process of the pancreas, using DW-MRI scans.

**Methods:**

We present and discuss 4 cases with neuroblastoma for a technical note. Imaging scans for SSR were performed using Ga68-DOTATATE PET/CT, and all showed varying degrees of increased uptake at the uncinate process of the pancreas on PET/CT images. We also performed a DW-MRI study to distinguish physiologic uptake in this region of the pancreas from metastatic involvement.

**Results:**

Two of them showed diffusion restriction, with one of them also showing multiple masses within the liver. The other 2 children with high pancreatic uncinate process uptake did not exhibit any findings that indicated pancreatic involvement in the disease, based on DW-MRI images and clinical findings.

**Conclusion:**

We recommend comparing DW-MRI scans and SSR-PET/CT scans to determine the true state of physiologically elevated SSR concentration and consequently indicate increased uptake on the images. The radiotracer concentration at the high uptake site did not appear to correlate with malignant involvement of the organ. The higher number of patients may allow a statistical comparison of the tracer with malignancy status.

## 1 INTRODUCTION

Neuroendocrine tumors express SSR on their cell surface. This will help image these tumor sites and treat them with appropriate radiolabeled markers such as Ga68-DOTA peptide molecules [1]. SSR imaging with Ga68-labeled molecules replaced In111-labeled octreotide scans due to the more suitable energy of the isotope and the superior imaging properties of PET/CT devices [2]. PET/CT offers superior details and a high target-to-background ratio at a comfortable speed compared to traditional gamma camera imaging with In111 [3]. Different compounds are being developed to map different subtypes of SSR subtypes (Table **1**) [4].

Pediatric patients with neuroblastoma were also imaged with Ga68-DOTA Peptid PET/CT to determine their tumor stage and decide on appropriate Lu177-labeled peptides for their treatment cycles if I123-MIBG scans were found negative [5]. Understanding the physiologic uptake sites and potential pitfalls is critical to help clinicians in delineating increased receptor expression sites [6]. The uncinate process of the pancreas is composed of more cells rich in SSR compared to other parts of this neuroendocrine gland [7]. Therefore, it appears on PET/CT images as a prominent uncinate process with high Ga68-DOTA peptide uptake. This can sometimes complicate the interpretation of images for the new lesion, particularly in children with left-sided adrenal tumors or abdominal-associated lesions. Pancreatic involvement in neuroectodermal tumors can sometimes be primary or metastatic [8, 9]. This is a clinically important situation. There are some strategies that might be used to differentiate physiological uptake from tumor uptake: (**1**). Comparison with anatomical imaging: Cross-referencing the PET/CT findings with high resolution CT or DW-MRI can help identify anatomical anomalies suggestive of a tumor. (**2**). Intensity of uptake: Tumors often show a higher intensity of uptake compared to physiological uptake. However, this is not always a definitive method, as some tumors may not be highly avid, and physiological uptake can sometimes be intense. (**3**). Patterns of uptake: Tumors may display a more focal pattern of uptake, while physiological uptake tends to be more uniform. Additionally, it's important to consider the patient's clinical history and other imaging findings when interpreting PET/CT scans. In some cases, biopsy or surgical exploration may be necessary for a conclusive diagnosis [10-12]. Neither physiological uptake nor metastasis should be overcome by PET/CT images of the Ga68-DOTA peptide alone. We presented 4 children diagnosed with neuroblastoma and imaged with Ga68-DOTATATE PET/CT and DW-MRI within 1 month to evaluate pancreatic invasion.

## 2 MATERIALS AND METHODS

Patients diagnosed with neuroblastoma between April 2020 and August 2022 were admitted to the PET/CT Center for a Ga68-DOTATATE PET/CT scan to determine their disease progression status. After undergoing an I123-MIBG scan they had negative or low uptake of their primary tumors. No fasting status is required for the exam. A whole-body PET/CT scan (Discovery IQ PET/CT, GE Medical Systems, USA) was performed with Ga68-DOTATATE. The radiopharmaceutical drug was synthesized from the eluted form of Ga68 using an in-house generator and administered intravenously to the children according to their weight (55.5-96.2 MBq mean: 74 MBq). PET/CT images were acquired 40-50 minutes after injection. Three patients were included in this technical note series. The children's ages are between 3-5 years (mean: 4.3). CT dose reduction with specific pediatric field of view adjustments was applied (CT dose index: 3.01 to 4.02 mGy-mean: 3.50 mGy). We performed DW-MRI primarily when high physiological uptake was detected at the uncinate process of the pancreas to aim for a differential diagnosis of tumor cells or normal pancreatic cell uptake in this part of the organ, especially in patients with advanced disease. Demographic data and dose information, as well as lesion sites of 4 children diagnosed with neuroblastoma, are summarized in Table **2**.

## 3 CASE SERIES

**Fig. (1):**
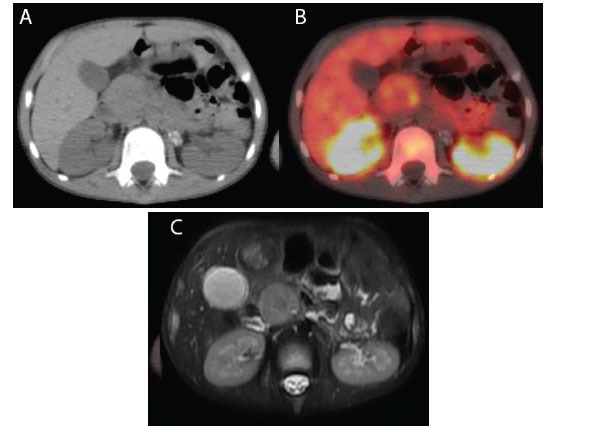
The uncinate process of the pancreas displays heterogeneous uptake and central relative low activity, as seen in the CT image (**A**) and corresponding Ga68-DOTATATE fusion image (**B**) of Case 1. Diffusion restriction and malignant involvement of the region are seen on the DW-MRI scan (**C**). In this instance, the pancreas was also involved in addition to the widespread illness. Following three months of imaging tests, he passed away.

**Fig. (2):**
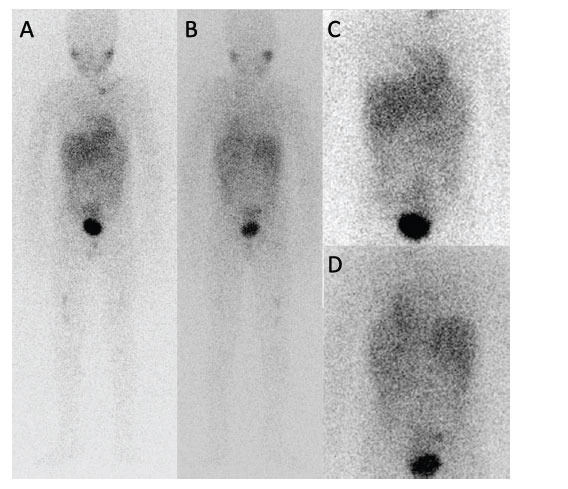
The I123-MIBG scan of Case 1 reveals metastatic bone uptake in the left and right distal femur, the right proximal tibia, and left clavicle. Conversely, there was no abnormal uptake in the abdomen, which does not align with the findings of the Ga68-DOTA peptide PET/CT scan. (**A**). Anterior whole-body scan (**B**). Posterior whole-body scan (**C** and **D**). Anterior and posterior thoracoabdominal static images.

**Case 1:** A 5-year-old boy was diagnosed with stage IV neuroblastoma of the left adrenal gland. He underwent surgery after chemotherapy. However, after 2 years of follow-up, there was a relapse of the disease. Disseminated bone marrow involvement and hyperactivity in the uncinate process location of the pancreatic head, as well as moderate Ga68-DOTATATE PET/CT uptake on the site, were noted (SUVMax: 6.49) (Fig. **1**). Because it is indistinguishable from normal appearance, a DW-MRI scan was performed, which showed a 32 mm lesion near the pancreatic head and the junction of the uncinate process. He was clinically thought to have pancreatic metastases in this region. The I123-MIBG scan showed no abnormal uptake at the pancreatic site, only bone metastases (Fig. **2**). The patient later died of disseminated disease.

**Fig. (3):**
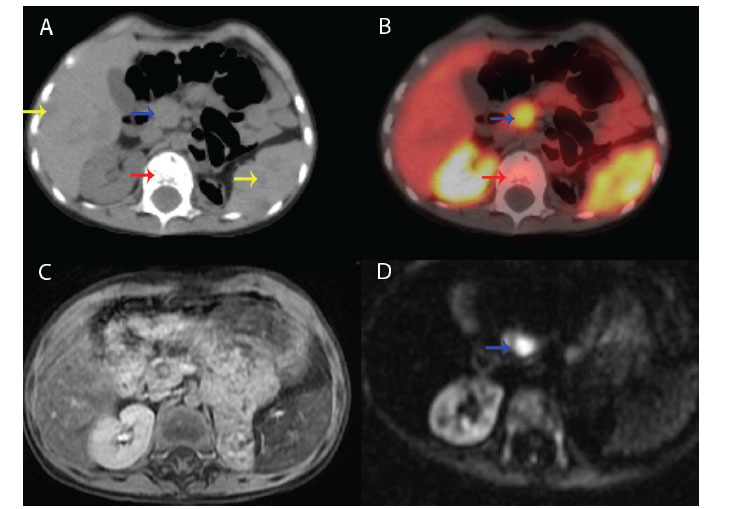
An uncinate process of the pancreas exhibits intense uptake, as seen in the CT image (**A**) and the Ga68-DOTATATE fusion image (**B**) of Case 2. T2-weighted (**C**) and diffusion-weighted images (**D**) also demonstrate the presence of a tumor that is causing diffusion restriction (blue arrows). Additionally, a small focus is seen on the organ's tail. The clinician was informed of multiple bone infiltrations (*i.e*., T12 vertebrae-red arrows) and liver/spleen metastases (yellow arrows) in addition to pancreatic involvement.

**Case 2:** A 5-year-old boy was diagnosed with stage IV neuroblastoma of the left adrenal gland. His lesion was surgically removed, and a bone marrow transplant was performed after chemotherapy. During his follow-up, a Ga68-DOTATATE PET/CT scan was performed, and pancreatic uncinate process activity (SUVMax: 8.17) and multiple bone marrow involvement with the disease were noted. CT images also revealed multiple liver and spleen metastases, which didn’t have apparent Ga68-DOTATATE activity over the background. DW-MRI was done to examine the pancreas for any metastases to this organ. DW-MRI showed a diffusion-restricted lesion measuring 15 × 10 mm in diameter, consisting of pancreatic tissue metastases (Fig. **3**).

**Fig. (4):**
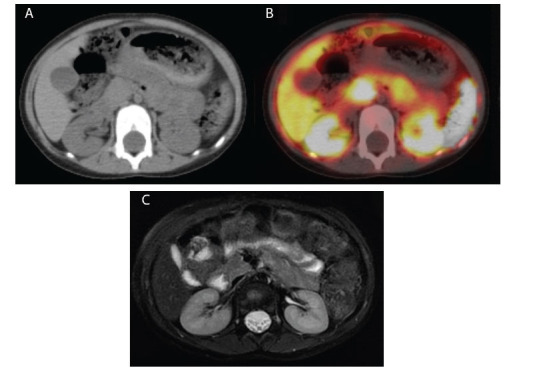
The CT scan (**A**) and matching Ga68-DOTATATE fusion image (**B**) of Case 3 demonstrate significant uptake on the pancreatic uncinate process. By using a diffusion-weighted MRI scan (**C**), which showed no abnormalities in the region, it was determined that the pancreatic region rich in SSR had physiologically high tracer uptake (SUVMax: 16.3).

**Case 3:** A 3-year-old girl was diagnosed with stage IV neuroblastoma of the right adrenal gland. Her tumor was shrunk with chemotherapy and then surgically removed. Her follow-up Ga68-DOTATATE PET/CT scan showed a prominent hypertrophic uncinate pancreatic flap and increased SSR uptake (SUVMax: 16.3). Two consecutive PET/CT scans showed the same findings and more uptake than usual visually in this part of the pancreas. The patient showed no signs of clinical recurrence. A DW-MRI scan was performed to clarify the primary lesion site and also the pancreas. The images showed no diffusion restriction at the uncinate process of the pancreas and were normal pancreatic tissue (Fig. **4**).

**Fig. (5):**
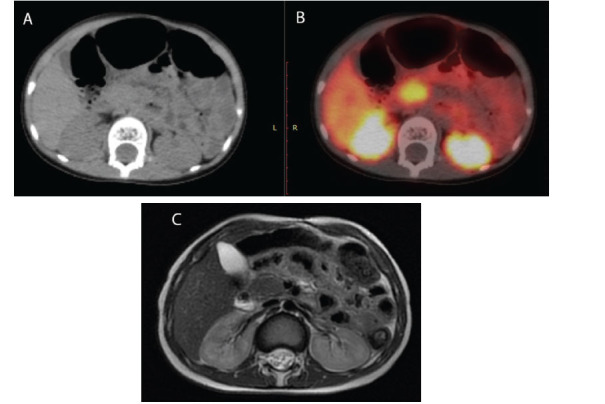
A 3-year-old boy was diagnosed with stage III neuroblastoma of the right adrenal gland (Case 4). The lesion was surgically removed and chemotherapy and radiotherapy were performed according to routine protocol. Hypertrophy of the pancreatic uncinate process and significant uptake on Ga68-DOTATATE PET/CT scan were noted (SUVMax: 7.75). It cannot be distinguished from physiological imaging and an MRI scan was performed. There were no diffusion abnormalities corresponding to the PET/CT scan site and the images were interpreted as normal appearance of the pancreatic tissue. The patient was followed up with no evidence of relapse. (**A**). CT scan (**B**). Ga68-DOTATATE fusion image (**C**). DW-MRI image.

**Case 4:** A 3-year-old boy was diagnosed with stage III neuroblastoma of the right adrenal gland. The lesion was surgically removed. Subsequent chemotherapy and radiotherapy were performed according to the routine protocol for the high-risk group during the consolidation phase of treatment after the induction phase. Hypertrophy of the pancreatic uncinate process and significant uptake on Ga68-DOTATATE PET/CT scan were noted (SUVMax: 7.75). It cannot be distinguished from physiological uptake, and a DW-MRI scan was performed. There were no diffusion abnormalities corresponding to the PET/CT scan site and the images were interpreted as normal appearance of the pancreatic tissue (Fig. **5**). The patient was followed up with no evidence of relapse.

## 4 RESULTS AND DISCUSSION

Pancreatic metastases were rare, particularly in children. Rhabdomyosarcoma, leukemia, lymphoma, and Ewing's sarcoma can sometimes lead to metastases to the pancreas in children [8, 13]. However, as imaging techniques are integrated into routine follow-up care for these patients, evidence of pancreatic involvement in neuroblastoma is becoming more common [14]. In order to identify SSR-positive tumor burden in pediatric neuroblastoma patients, the Ga68-DOTA peptide PET/CT study is currently used in addition to the I123-MIBG scan when available. This is because the data from the SSR scan is used to guide the administration of Lu177-DOTA peptide treatment after traditional chemotherapy and radiation therapy [15-19]. For an accurate assessment of disease burden, especially in the vicinity of physiological tracer uptake sites, an accurate interpretation of the Ga68-DOTA peptide scan is essential [6]. The differentiation of physiological and tumor uptake in the uncinate process of the pancreas when using Ga68-DOTA peptide PET/CT imaging in children with neuroblastoma can sometimes be challenging. The uncinate process of the pancreas is one of these physiological uptake sites for the Ga68-DOTA peptide, and it varies substantially between patients based on the number of SSR-containing cells in the organ. Due to the inconsistent presentation of findings amongst patients, fluctuations in these physiological uptake sites can lead to both false positive and false negative interpretations. Pancreatic invasion of neuroblastoma can sometimes be observed throughout the entire duration of the disease and makes it difficult to distinguish from normal situations [20]. All four of our cases underwent Ga68-DOTATATE scans, and in two of them, DW-MRI and clinical findings confirmed the presence of pancreatic metastases. One of the patients with metastases (Case 1) had an I123-MIBG scan performed, which showed no metastases in this region. However, this MIBG scan showed bone marrow involvement in the lower extremity. When it comes to the tumor's receptor expression, the cellular components of the metastases may differ. The other child with pancreatic metastases (Case 2) also did not exhibit any uptake at the uncinate process of the pancreas on I123-MIBG scans. The other two patients (Cases 3 and 4) also showed varying degrees of SSR uptake (SUVMax: 16.3 and 7.75, respectively) at the uncinate process despite normal patterns on DW-MRI. These findings imply that since SSR expression in the pancreatic uncinate process can vary, malignant involvement of this organ cannot be predicted based on SUVMax values of Ga68-DOTATATE uptake. However, focal activity involvement in the uncinate process, which significantly exceeds the liver activity in high-risk neuroblastoma patients, diffusion MRI imaging of this region, particularly in patients without other metastatic lesions, may be crucial in terms of altering the treatment plan. If elevated tracer uptake is noted at the pancreatic uncinate process, we advise comparing the interpretation of the Ga68-DOTA peptide PET/CT scan with DW-MRI scans [3, 9, 18]. It is appropriate to classify this uptake as pathologic tracer accumulation when diffusion restriction is observed in the relevant pancreatic region. The clinician can alter the treatment plan to a more aggressive alternative after receiving this crucial information. The extent of the disease can also be ascertained by combining I123-MIBG scintigraphy with SPECT/CT imaging of the affected area, which is primarily the abdomen [21-23]. However, the cellular characteristics of patients may vary from patient to patient, and while some children may have a mass with MIBG avid lesions, others may only have avid cells of the SSR or both. This makes PET/CT scanning of Ga68-DOTA peptides an important step in the follow-up of children with neuroblastoma. The level of receptor expression determined by SUVMax values appears to be independent of organ infiltration status [5, 24, 25].

## CONCLUSION

Based on the cases presented here, we strongly recommend comparing the two modalities when interpreting high tracer uptake at physiological sites to determine whether it represents a true pathological site or a common physiological finding. The limitation of this study is that it is based on observations in a small patient population. Future studies with larger cohort series are needed to verify the findings.

## Figures and Tables

**Table 1:** SSR Analogs for PET/CT imaging.

**Compound**	**Receptor Subtypes**
Ga68-DOTATATE (GaTate)	STR 2
Ga68-DOTANOC (GaNoc)	STR 3, SSTR 5
Ga68-DOTATOC (GaToc)	STR 5

**Table 2:** Patients’ demographic and lesion information.

**-**	**Age**	**M/F**	**Side**	**SUVMax**	**DW-MRI**
Case 1	5	M	Left	6.49	Metastasis
Case 2	5	M	Left	8.17	Metastasis
Case 3	3	F	Right	16.3	N
Case 4	3	M	Right	7.75	N

## Data Availability

All the data and supporting information is provided within the article.

## LIST OF ABBREVIATIONS

DW-MRI: Diffusion-Weighted Magnetic Resonance Imaging
Ga68: Gallium-68
I123: Iodine-123
In111: Indium-111
Lu177: Lutetium-177
MIBG: Meta-Iodo Benzyl Guanidine
PET/CT: Positron Emission Tomography/Computeri sed Tomography
SSR: Somatostatin Receptors
SUVMax: Standardised Uptake Value

## STANDARDS OF REPORTING

STROBE guidelines were followed.

## References

[1] O’Dorisio M.S., Khanna G., Bushnell D. (2008). Combining anatomic and molecularly targeted imaging in the diagnosis and surveillance of embryonal tumors of the nervous and endocrine systems in children.. Cancer Metastasis Rev..

[2] Bar-Sever Z., Biassoni L., Shulkin B., Kong G., Hofman M.S., Lopci E., Manea I., Koziorowski J., Castellani R., Boubaker A., Lambert B., Pfluger T., Nadel H., Sharp S., Giammarile F. (2018). Guidelines on nuclear medicine imaging in neuroblastoma.. Eur. J. Nucl. Med. Mol. Imaging.

[3] Hofman M.S., Lau W.F.E., Hicks R.J. (2015). Somatostatin receptor imaging with 68Ga DOTATATE PET/CT: Clinical utility, normal patterns, pearls, and pitfalls in interpretation.. Radiographics.

[4] Kroiss A., Putzer D., Decristoforo C., Uprimny C., Warwitz B., Nilica B., Gabriel M., Kendler D., Waitz D., Widmann G., Virgolini I.J. (2013). 68Ga-DOTA-TOC uptake in neuroendocrine tumour and healthy tissue: Differentiation of physiological uptake and pathological processes in PET/CT.. Eur. J. Nucl. Med. Mol. Imaging.

[5] Gains J.E., Aldridge M.D., Mattoli M.V., Bomanji J.B., Biassoni L., Shankar A., Gaze M.N. (2020). 68Ga-DOTATATE and 123I-mIBG as imaging biomarkers of disease localisation in metastatic neuroblastoma: Implications for molecular radiotherapy.. Nucl. Med. Commun..

[6] Krausz Y., Rubinstein R., Appelbaum L., Mishani E., Orevi M., Fraenkel M., Tshori S., Glaser B., Bocher M., Salmon A., Chisin R., Gross D.J., Freedman N. (2012). Ga-68 DOTA-NOC uptake in the pancreas: Pathological and physiological patterns.. Clin. Nucl. Med..

[7] Tabacchi E., Fortunati E., Argalia G., Zanoni L., Calabrò D., Telo S., Campana D., Lamberti G., Ricci C., Casadei R., Fanti S., Ambrosini V. (2022). [68Ga]Ga-DOTANOC uptake at pancreatic head/uncinate process: Is it a persistent diagnostic pitfall over time?. Cancers.

[8] Raymond S.L.T., Yugawa D., Chang K.H.F., Ena B., Tauchi-Nishi P.S. (2017). Metastatic neoplasms to the pancreas diagnosed by fine-needle aspiration/biopsy cytology: A 15-year retrospective analysis.. Diagn. Cytopathol..

[9] Shi L., Guo Z., Wu X. (2013). Primary pulmonary primitive neuroectodermal tumor metastasis to the pancreas: A rare case with seven-year follow-up.. Diagn. Pathol..

[10] Jacobsson H., Larsson P., Jonsson C., Jussing E., Grybäck P. (2012). Normal uptake of 68Ga-DOTA-TOC by the pancreas uncinate process mimicking malignancy at somatostatin receptor PET.. Clin. Nucl. Med..

[11] Kong G., Hofman M.S., Murray W.K., Wilson S., Wood P., Downie P., Super L., Hogg A., Eu P., Hicks R.J. (2016). Initial experience with Gallium-68 DOTA-Octreotate PET/CT and peptide receptor radionuclide therapy for pediatric patients with refractory metastatic neuroblastoma.. J. Pediatr. Hematol. Oncol..

[12] Fathpour G., Jafari E., Hashemi A., Dadgar H., Shahriari M., Zareifar S., Jenabzade A.R., Vali R., Ahmadzadehfar H., Assadi M. (2021). Feasibility and therapeutic potential of combined peptide receptor radionuclide therapy with intensive chemotherapy for pediatric patients with relapsed or refractory metastatic neuroblastoma.. Clin. Nucl. Med..

[13] Jha P., Frölich A.M.J., McCarville B., Navarro O.M., Babyn P., Goldsby R., Daldrup-Link H. (2010). Unusual association of alveolar rhabdomyosarcoma with pancreatic metastasis: Emerging role of PET-CT in tumor staging.. Pediatr. Radiol..

[14] Shukla J., Goel R., Bansal D., Sodhi K., Bhattacharya A., Marwaha R., Mittal B. (2014). 68 Ga-DOTATATE positron emission tomography/computed tomography scan in the detection of bone metastases in pediatric neuroendocrine tumors.. Indian J. Nucl. Med..

[15] Sundquist F., Georgantzi K., Jarvis K.B., Brok J., Koskenvuo M., Rascon J., van Noesel M., Grybäck P., Nilsson J., Braat A., Sundin M., Wessman S., Herold N., Hjorth L., Kogner P., Granberg D., Gaze M., Stenman J. (2022). A phase II trial of a personalized, dose-intense administration schedule of 177Lutetium-DOTATATE in children with primary refractory or relapsed high-risk neuroblastoma–LuDO-N.. Front Pediatr..

[16] Koo S.X., Tong A.K., Soh S.Y., Farid M., Loh A., Loke K.S. (2021). Selective use of peptide receptor radionuclide therapy following comparative imaging of Ga-68 DOTATATE PET/CT against I-131 MIBG scintigraphy in a small asian cohort of adult neuroblastoma.. Med. J. Malaysia.

[17] Gains J.E., Moroz V., Aldridge M.D., Wan S., Wheatley K., Laidler J., Peet C., Bomanji J.B., Gaze M.N. (2020). A phase IIa trial of molecular radiotherapy with 177-lutetium DOTATATE in children with primary refractory or relapsed high-risk neuroblastoma.. Eur. J. Nucl. Med. Mol. Imaging.

[18] Alexander N., Vali R., Ahmadzadehfar H., Shammas A., Baruchel S. (2018). Review: The role of radiolabeled DOTA-conjugated peptides for imaging and treatment of childhood neuroblastoma.. Curr. Radiopharm..

[19] Gains J.E., Bomanji J.B., Fersht N.L., Sullivan T., D’Souza D., Sullivan K.P., Aldridge M., Waddington W., Gaze M.N. (2011). 77Lu-DOTATATE molecular radiotherapy for childhood neuroblastoma.. J. Nucl. Med..

[20] Kim E.Y., Yoo S.Y., Kim J.H., Sung K.W. (2008). Pancreatic metastasis in a child suffering with treated stage 4 neuroblastoma.. Korean J. Radiol..

[21] Biassoni L., Privitera L. (2021). 123I-meta-iodobenzylguanidine single-photon emission computerized tomography/computerized tomography scintigraphy in the management of neuroblastoma.. Indian J. Nucl. Med..

[22] Sharp S.E., Trout A.T., Weiss B.D., Gelfand M.J. (2016). MIBG in neuroblastoma diagnostic imaging and therapy.. Radiographics.

[23] Bleeker G., Tytgat G.A.M., Adam J.A., Caron H.N., Kremer L.C.M., Hooft L., van Dalen E.C. (2015). 123I-MIBG scintigraphy and 18F-FDG-PET imaging for diagnosing neuroblastoma.. Cochrane Libr..

[24] McElroy K.M., Binkovitz L.A., Trout A.T., Czachowski M.R., Seghers V.J., Lteif A.N., States L.J. (2020). Pediatric applications of Dotatate: Early diagnostic and therapeutic experience.. Pediatr. Radiol..

[25] Bodei L., Ambrosini V., Herrmann K., Modlin I. (2017). Current concepts in 68 Ga-DOTATATE imaging of neuroendocrine neoplasms: Interpretation, biodistribution, dosimetry, and molecular strategies.. J. Nucl. Med..

